# Paper Stacks for Uniform Rehydration of Dried Reagents in Paper Microfluidic Devices

**DOI:** 10.1038/s41598-019-52202-9

**Published:** 2019-10-31

**Authors:** Debayan Das, Andrea Dsouza, Navjot Kaur, Shruti Soni, Bhushan J. Toley

**Affiliations:** Department of Chemical Engineering, Indian Institute of Science Bangalore, Bangalore, 560012 India

**Keywords:** Biomedical engineering, Tuberculosis, Fluid dynamics

## Abstract

Spatially uniform reconstitution of dried reagents is critical to the function of paper microfluidic devices. Advancing fluid fronts in paper microfluidic devices drive (convect) and concentrate rehydrated reagents to the edges, causing steep chemical gradients and imperfect mixing. This largely unsolved problem in paper microfluidics is exacerbated by increasing device dimensions. In this article, we demonstrate that mixing of dried reagents with a rehydrating fluid in paper microfluidics may be significantly enhanced by stacking paper layers having different wicking rates. Compared to single-layer paper membranes, stacking reduced the “non-reactive area”, i.e. area in which the reconstituted reagents did not interact with the rehydrating fluid, by as much as 97% in large (8 cm × 2 cm) paper membranes. A paper stack was designed to collect ~0.9 ml liquid sample and uniformly mix it with dried reagents. Applications of this technology are demonstrated in two areas: (i) collection and dry storage of sputum samples for tuberculosis testing, and (ii) salivary glucose detection using an enzymatic assay and colorimetric readout. Maximizing the interaction of liquids with dried reagents is central to enhancing the performance of all paper microfluidic devices; this technique is therefore likely to find important applications in paper microfluidics.

## Introduction

Paper microfluidic devices have become an important platform for conducting analytical chemistry, especially for applications in point-of-care diagnostics^1,2^. Paper (or porous membranes in general) is used as a substrate in many types of analytical devices, e.g. lateral flow assays (LFAs), multiplexed colorimetric assays (µPADs)^3^, two-dimensional paper networks (2DPNs)^4^, paper ELISA (P-ELISA)^5^, and paper-based nucleic acid amplification tests (P-NAATs)^6^, among several others. A critical operation in all these devices is the rehydration of reagents stored dry in a porous membrane, e.g. gold-antibody conjugates are stored dry in LFAs, enzymes are stored dry in µPADs etc. Spatially uniform rehydration and sufficient mixing of the dry-stored reagents with the rehydrating fluid (often the sample used for analysis) is therefore critical for efficient and reproducible performance of the analytical device. For example, it has been shown that non-uniform rehydration leads to variable performance and heterogeneous color development in µPADs, which can lead to poor judgement of signal readout by the user^7^.

When a fluid is introduced into a paper membrane, the pores within the paper induce a capillary force on it that causes the fluid to wick. If the pores contain dried reagents, they dissolve in this process and move (convect) along the moving fluid front. If the dissolution is rapid, most of the rehydrated reagents get pushed along with the fluid front to regions farther away from the fluid source and get concentrated near the edges, thus reducing the interaction between the dried reagents and the fluid entering the paper. This compaction of reagents is well demonstrated by Lutz *et al*.^8^ and is exacerbated as the length of the paper increases. After the reagents are compacted at the fluid front, it is not possible to mix these with the lagging fluid because of low-Reynolds-number laminar flows and the inability to stir. Some methods have been developed to enhance mixing in paper microfluidics. Osborn *et al*. demonstrated that mixing of two colored dye solutions could be enhanced by stacking two paper membranes^9^. Rezk *et al*. demonstrated enhanced mixing using surface acoustic waves^10^. However, both these techniques were developed to enhance mixing between two liquids. The challenge of enhancing mixing between a dry-stored reagent and a rehydrating fluid is largely unsolved in paper microfluidics.

In this article, we demonstrate a technique specifically designed to enhance mixing between reagents dried in paper microfluidic membranes and a rehydrating fluid. This is accomplished by stacking two paper membranes having significantly different wicking rates. The slow-wicking (high-resistance) membrane is used to store dried reagents and the fast-wicking (low-resistance) membrane is used as a fluid distributor. In comparison to a single-layer paper membrane, this combination of materials a) significantly reduces gradients in concentration of rehydrated reagents across the dimensions of the device, b) increases volumetric capacity of the device, and c) reduces the time for rehydration. This technique has been developed as a part of a project funded by the Bill & Melinda Gates Foundation focused on developing a method to collect and dry (stabilize) sputum specimens in paper-based devices for downstream detection of tuberculosis. To accomplish this, it was necessary to enhance the volumetric capacity of paper-based devices well beyond their current capacity while ensuring that the sputum sample rapidly spread and effectively mixed with the stabilization chemistry dried in the device. Using paper stacks, we demonstrate a device that can store and effectively decontaminate 895 µl of a mock sputum sample by killing all non-*Mycobacterial* bacterial strains within the sputum. Single-layer paper devices could not accomplish this because of inadequate mixing. To demonstrate the versatility of this approach, we also demonstrate how paper stacks can be used to enhance the sensitivity of a paper-based bienzymatic glucose detection assay. This work presents a new approach for achieving uniform rehydration of dried reagents, critical to all paper-based analytical and stabilization devices.

## Results

### Surface distributors

**Figure 1:**
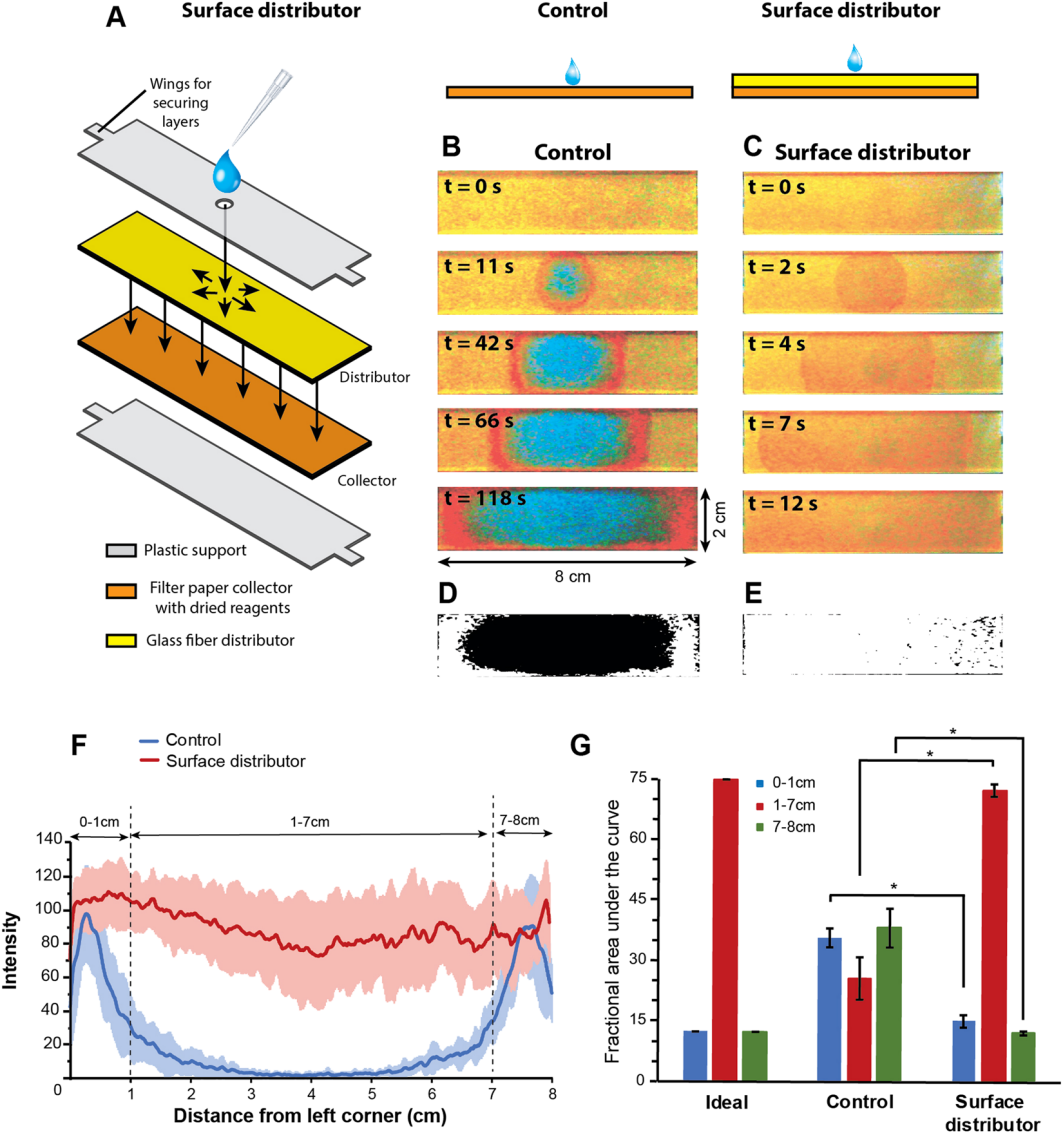
Surface distributors. (**A**) The surface distributor spreads fluid over a distributor placed directly on top of the collector of equal dimensions. (**B)** Fluid entering a single layer filter paper membrane (control) rehydrates the dried orange dye and pushes it to the edges. (**C**) The surface distributor eliminates migration of rehydrated reagents towards the edges. (**D**,**E**) Thresholded images showing areas containing orange dye as white and areas lacking orange dye (non-reactive areas) as black for control (**D**) and the surface distributor (**E**). The surface distributor significantly reduced the non-reactive area (P < 0.001; N = 3), nearly eliminating it. (**F**) Linear intensity profiles along the length show peaks in 0–1 cm and 7–8 cm for controls, but a flat profile for surface distributors. (**G**) Fractional areas under the linear intensity profile plots for a hypothetical case of ideal rehydration, controls, and surface distributors. The fractional area signature for surface distributors resembled ideal rehydration. Fractional areas under the curve for 0–1, 1–7, and 7–8 cm for surface distributors were significantly different from controls (*P < 0.001; N = 3).

**Figure 2:**
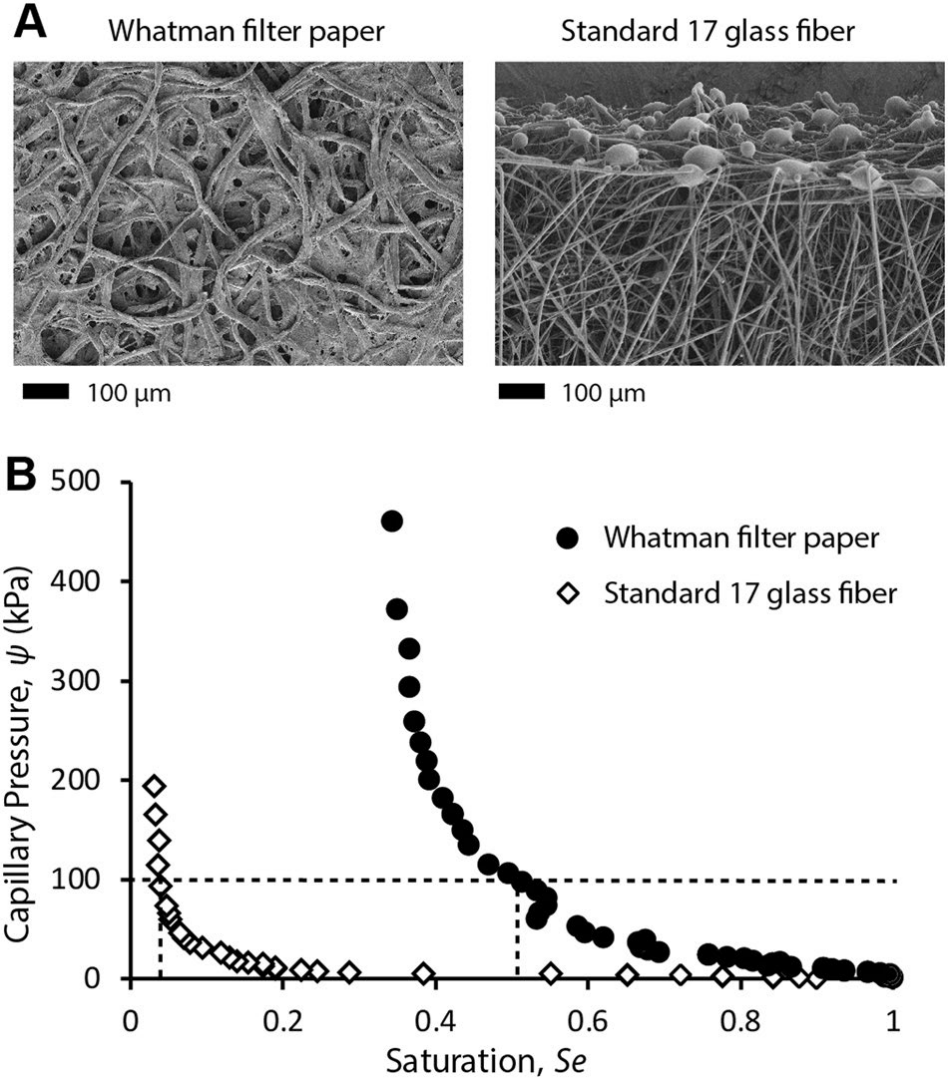
Characterization of distributor and collector materials. (**A**) Scanning electron microscopy images of the collector (Whatman filter paper) and distributor (Standard 17 glass fiber) materials. (**B**) Capillary pressure as a function of saturation (fraction of fluid-filled pores) for the collector and distributor materials.

In conventional paper microfluidic devices, fluids wick into regions containing dried reagents from a single inlet point. We hypothesized that increasing the number of inlet points would reduce the distance over which rehydrated reagents could be displaced and enhance interaction between the dried reagents and rehydrating fluid. Surface distributors accomplish this by creating a continuous fluid distribution surface by placing a paper distributor layer (yellow; Fig. 1A) directly on top of a paper collector layer containing dried reagents (orange; Fig. 1A). For this design, it is critical to choose the distributor and collector materials such that they differ significantly in wicking rates; specifically, the distributor layer must have a significantly higher wicking rate than the collector layer. Because the distributor wets rapidly, it provides fluid to the collector layer uniformly over the entire surface, eliminating lateral movement of rehydrated reagents in the collector. In the specific implementation in Fig. 1A, the distributor layer was constructed from Standard 17 glass fiber and the collector from Whatman filter paper. The top and bottom plastic layers were constructed from acrylic sheets and the layers were secured by wrapping adhesive tape around wings extending from their two edges (Fig. 1A). The parameter, *T*_*4-cm*_, defined as the time required for a fluid front to wick 4 cm away from the source in a rectangular strip of a given material, was measured to be 11.3 ± 1.15 s and 110 ± 13 s for Standard 17 and Whatman filter paper, respectively, showing significant difference in wicking rates. Typically, commercial diagnostic membranes with faster wicking rates have larger pore sizes and lower capillary pressure compared to slower wicking membranes that have smaller pore sizes and higher capillary pressure. Scanning electron microscopy images (Fig. 2A) and curves for capillary pressure vs saturation (*Se*, fraction of fluid-filled pores) for the two membranes (Fig. 2B) confirm this - only about 3% of pores in Standard 17 (*Se* ~ 0.03) have a capillary pressure above 100 kPA, whereas about 50% of pores in Whatman filter paper (*Se* ~ 0.5) have capillary pressure above 100 kPA. The lower capillary pressure of Standard 17 compared to Whatman filter paper also makes it a suitable choice for a distributor because it can effectively release fluid into the filter paper collector membrane.

Rehydration of a dried colored dye using surface distributors was compared to single-layer paper membranes (controls). The devices consisted of large (8 cm × 2 cm) filter paper membranes impregnated with dried food coloring dye. Detailed dimensions of the device are provided in Supplementary Fig. S1 and S2. The control, which contained only a single filter paper membrane, was rehydrated with 255 µl DI water corresponding to the fluidic capacity of the membrane. Fluid added at the center of the membrane wicked into the membrane in all directions away from the source. As the fluid front advanced, the dye dissolved in the water and moved along with the front (Fig. 1B). The membrane was fully wet at 118 s, at which point, a significant portion of the dye was pushed to the edges producing a large island devoid of the dye (Fig. 1B). For the surface distributor, 895 µl DI water was used for rehydration to accommodate for the capacity of the stacked membranes. The surface distributor accomplished a uniform and homogeneous distribution of the rehydrated dye over the entire surface (Fig. 1C). It further reduced the time of rehydration to 12 s because of the very low resistance to flow in the wide glass fiber distributor and rapid wicking from the distributor to the collector in the vertical direction (these phenomena are explained in detail in the section entitled, ‘Convective time scales in surface distributors’). Rapid movement of the fluid front away from the fluid source was observed in the surface distributor (Fig. 1C), but this movement was not associated with movement of rehydrated dye towards the edges. Regions devoid of dye correspond to regions where the rehydrating fluid cannot interact with the dried reagents (dye) and constitute a ‘non-reactive’ area. Therefore, a better rehydrating strategy would be the one that minimizes the non-reactive area or enhances the ‘reactive area’. The percentage non-reactive area estimated by thresholding the end-point images (Fig. 1D,E) reduced from a mean value of 70.4% for controls to 1.6% for surface distributors (>97% reduction; P < 0.001; N = 3). The interaction between the rehydrating fluid and dried reagents was thus significantly enhanced by the surface distributor. In additional experiments, an alternate distributor design motivated by multi-layer three-dimensional paper analytical devices (3D µPADs), which are devices designed to distribute a given fluid volume into multiple analytical zones^11,12^, was also tested for its ability to enhance the interaction between dried reagents and the rehydrating fluid. This “multi-inlet distributor” reduced the non-reactive area from 70.4% to 46.7% (P < 0.005; N = 3) and therefore significantly underperformed compared to surface distributors (see Supplementary Fig. S3).

To further analyze the distribution of rehydrated dye in surface distributors, linear intensity profiles were obtained for controls and surface distributors along their 8-cm length (Fig. 1F). For controls, there were two distinct peaks in between 0–1 cm and 1–8 cm and very low intensities in between 1–7 cm (blue line; Fig. 1F). This signifies that most of the rehydrated dye was pushed away to the sides of the membrane. In comparison, for surface distributors, the intensity plots were relatively flat without any distinct peaks (red line; Fig. 1F). For statistical comparison, areas under the linear intensity profiles were calculated for 0–1, 1–7, and 7–8 cm in addition to the total area (0–8 cm). In a hypothetical ideal case of uniform rehydration, the linear intensity profile would be a horizontal line. In such a case, fractional areas under the curve for 0–1, 1–7, and 7–8 cm would be 12.5–75–12.5% (Fig. 1G). This can be considered a signature of ideal performance for benchmarking. For controls, the corresponding rehydration signature was 36–26–38% (mean; N = 3; Fig. 1G) and for surface distributors was 15–73–12% (mean; N = 3; Fig. 1G) – the latter strongly matching the signature for ideal performance. Further, the 0–1, 1–7, and 7–8 cm fractional areas under the curve for surface distributors were significantly different than those for controls (*P < 0.001; N = 3; Fig. 1G). Surface distributors, therefore, significantly improved the uniformity of dried reagent rehydration.

In addition to the above quantitative analysis, the concentration of the rehydrated dye could be semi-quantitatively inferred from its color. A clear shift from yellow to red (darkening) was observed after rehydration in controls (Fig. 1B and Supplementary Fig. S3) and some red regions were obtained using “multi-inlet distributors” (Supplementary Fig. S3). This darkening is an effect of concentration of the rehydrated reagent. There was, however, a distinct lack of darkening when surface distributors were used for rehydration (Fig. 1C). This shows that surface distributors are effective in maintaining spatially uniform concentration of reagents during the process of rehydration. This may be critical for certain analytical applications requiring an optimum reagent concentration range. For example, very high concentration of DNA primers can lead to formation of primer-dimers in nucleic acid amplification reactions^6^ while very low concentrations may lead to inefficient/slow reactions.

### Convective time scales in surface distributors

**Figure 3:**
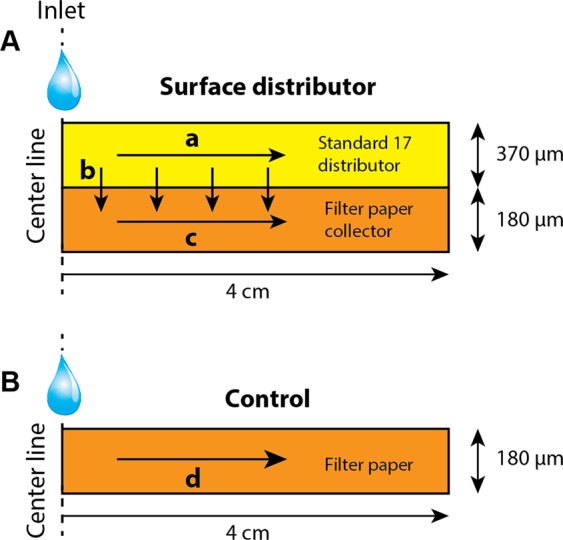
Convective flow in surface distributors. Fluid entering the surface distributor can wick into the distributor (direction a), wick into the collector (direction b), and the fluid that reaches the collector can wick in the collector away from the center (direction c). In the control, there is only one direction for wicking (direction d).

In this section, the rationale behind uniform rehydration enabled by the surface distributor is elucidated using various convective time scales. The movement of a fluid front during wicking in paper is known to follow Washburn behavior, according to which $L=K\sqrt{t}$, where *L* is distance of the fluid front from the source and *t* is time. Using values of *T*_*4-cm*_, the Washburn coefficient, *K*, for filter paper and glass fiber were determined to be 0.38 and 1.19 cm/s^0.5^_,_ respectively. Because the surface distributor in Fig. 1 is symmetric around the central inlet point, let us consider only the part of the device to the right of the inlet (Fig. 3). Fluid entering the distributor layer can wick in three different directions: in the horizontal direction in the distributor (a; Fig. 3A), in the vertical direction from the distributor to the collector (b; Fig. 3A), and in the horizontal direction in the collector (c; Fig. 3A). The time scales for convection in the three directions may be denoted by *t*_*a*_, *t*_*b*_, and *t*_*c*_. For directions ‘a’ and ‘c’, because the convection distance is 4 cm, the time scales are equivalent to the corresponding values of T_*4-cm*_, i.e. *t*_*a*_ = 11.3 s and *t*_*c*_ = 110 s, respectively. The time scale for convection in the vertical direction ‘b’ can be calculated as the time required for fluid to wick through 180 µm length of filter paper. Using the Washburn coefficient for filter paper, *t*_*b*_ was estimated to be 2.43 ms. The following can be concluded from these time scale: (i) fluid entering the distributor rapidly saturates the distributor (within 11.3 s), (ii) while the fluid wicks into the distributor in the direction ‘a’, it immediately (within 2.43 ms) wets the collector under it, and (iii) once fluid wicks vertically into the collector, the rate at which it wicks in direction ‘c’ is significantly slower (of the order of 110 s). In fact, once fluid reaches the collector, it remains stagnant because the rate of vertical rehydration of the collector through the distributor is significantly higher than the rate at which the collector would wick fluid in the direction ‘c’. Therefore, the dominant mechanism of wetting the collector is wicking of fluid from the distributor in the vertical direction ‘b’. Because fluid in the filter paper remains stagnant, rehydrated reagents in the filter paper do not move, resulting in uniform rehydration. It is apparent that a large difference in wicking rates (*T*_*4-cm*_) between the distributor and collector layers is critical for uniform rehydration. On the other hand, in the corresponding control that lacks a distributor (Fig. 3B), there is only one convective time scale, *t*_*d*_ = *t*_*c*_ = 110 s, and the fluid entering the filter paper must wick through regions containing dried reagents and displace rehydrated reagents to the edges.

### Effect of collector material

The possibility of using materials other than filter paper as the collector was briefly explored using two alternatives – nitrocellulose (NC) FF120 and Standard 17 glass fiber. In both cases, the distributors were made of Standard 17 glass fiber and controls (without distributors) were used for direct comparison. The combination of Standard 17 (*T*_*4-cm*_ = 11.3 s ± 1.15 s) and NC FF120 (*T*_*4-cm*_ = 148 s ± 10 s) was very effective for uniform rehydration of reagents stored in the NC, whereas when both distributor and collector were made of Standard 17, the rehydration was not uniform (see Supplementary Fig. S4). This observation is consistent with the requirement of significantly different wicking rates between the distributor and collector, which was violated for the latter case.

### Compatibility with viscous samples

**Figure 4:**
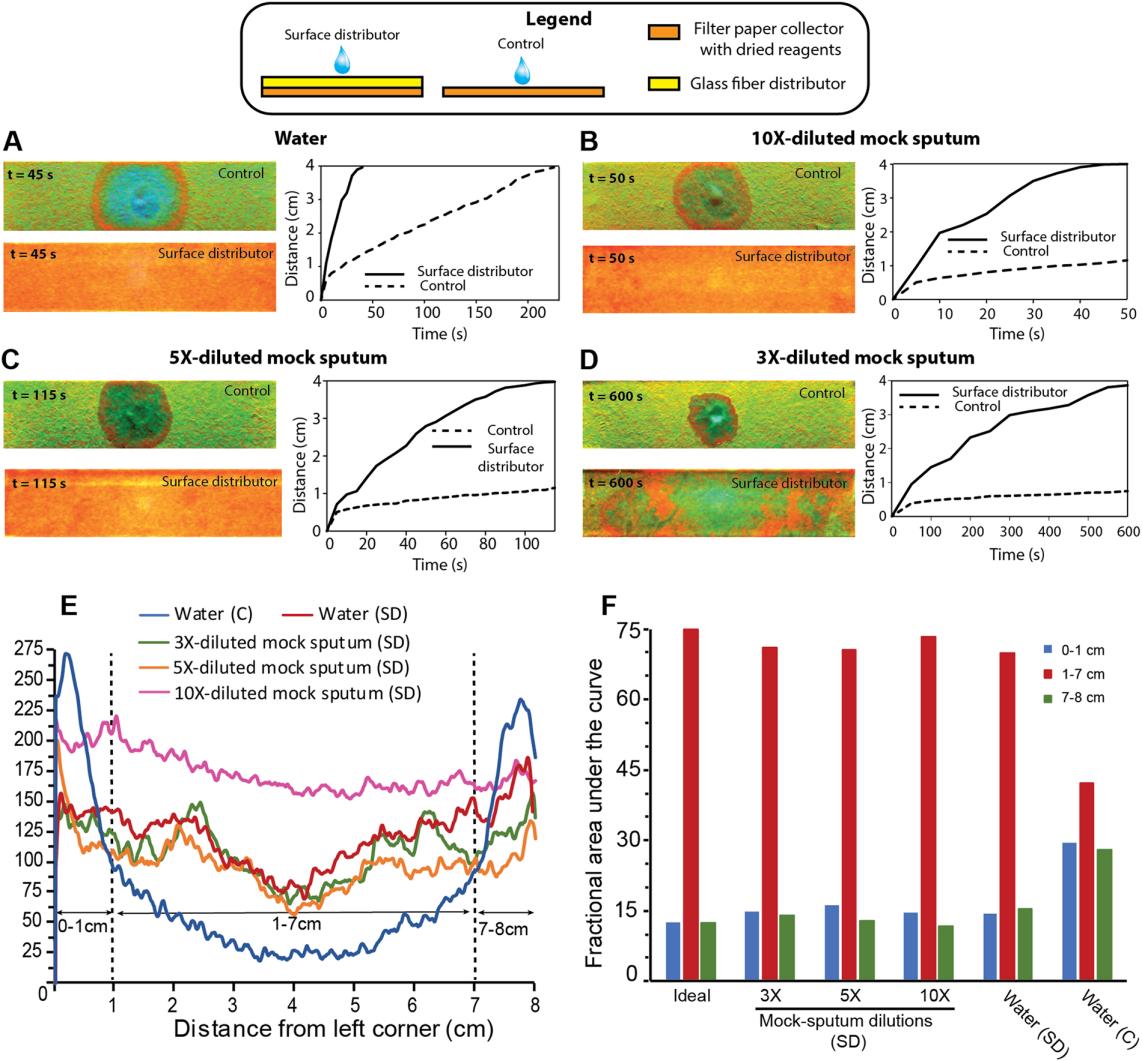
Flow of mock sputum through paper stacks. (**A–D**) Images of controls and surface distributors at time points at which the entire 8-cm length of surface distributors had just been traversed by the fluid and the corresponding plots for distance of fluid fronts from the point of fluid introduction over time, for water (**A**), 10 × -diluted mock sputum (**B**), 5 × -diluted mock sputum (**C**), and 3 × -diluted mock sputum (**D**). Surface distributors significantly enhanced the rate of wicking. (**E**) Linear intensity profiles along 8-cm lengths. C: control (single layer Whatman filter paper); SD: surface distributor. Despite the viscosity of mock sputum, intensity profiles were flatter for SD compared to water flowed in C, indicating uniform rehydration in SD. (**F**) Fractional areas under the linear intensity profile plots for 0–1, 1–7, and 7–8 cm regions. For all dilutions of mock sputum and for water introduced into SD, the area under the curve signature resembled that of ideal rehydration. For C, the signature was far from ideal.

Clinical specimens may be more viscous than DI water and therefore it was important to verify the compatibility of surface distributors with more viscous samples. Because this work was conducted in the context of sputum stabilization, different dilutions of mock sputum (undiluted viscosity = 350 cP) were introduced into surface distributors and controls (all of size 8 cm × 2 cm). The four fluids used for rehydration were 3X, 5X, and 10X-diluted mock sputum and DI water. Surface distributors were rehydrated with 895 µl and controls with 255 µl of the corresponding rehydrating fluid, corresponding to their absorption capacities. The distance between the point of fluid introduction (center) and the advancing fluid front on one side over time, and linear intensity profiles over 8-cm lengths after fluid fronts traversed the full length of devices were evaluated. In all cases, surface distributors enhanced the rate of wicking. For water, 10X-, 5X-, and 3X-diluted mock sputum, the surface distributor devices were fully rehydrated at 45, 50, 115 and 600 s, respectively (Fig. 4A–D); whereas for the corresponding controls, only water fully rehydrated the device at 230 s – none of the diluted mock sputum solutions reached the end of the device within the observation period (Fig. 4A–D). This demonstrates that by providing a low-resistance path to flow, surface distributors increase the compatibility of paper-based devices with viscous samples.

An analysis of the uniformity of the rehydrated orange dye was further performed. Visual analysis revealed that for water, 10X- and 5X-diluted mock sputum, surface distributors resulted in homogenously uniform rehydration (Fig. 4A–C). For 3X-diluted sputum, which was the most viscous sample tested, the uniformity was less than ideal, and some yellow and orange patches were observed (Fig. 4D). Linear intensity profiles over 8-cm lengths for all conditions are shown in Fig. 4E. Distinct peaks within 0–1 and 7–8 cm were again observed for the control rehydrated with water (blue curve; Fig. 4E). However, there was a distinct lack of such peaks in the profiles of surface distributors rehydrated with all four fluids (Fig. 4E). The 0–1, 1–7, and 7–8 cm fractional area under the curve signature for surface distributors rehydrated with all four fluids matched the ideal signature (12.5–75–12.5%) very well (Fig. 4F), whereas for the control rehydrated with water, there was a marked deviation from the ideal signature (Fig. 4F; rightmost condition). These results show that despite high viscosity of samples, surface distributors enable spatially uniform reagent rehydration.

### Bacterial decontamination in paper stacks

**Figure 5:**
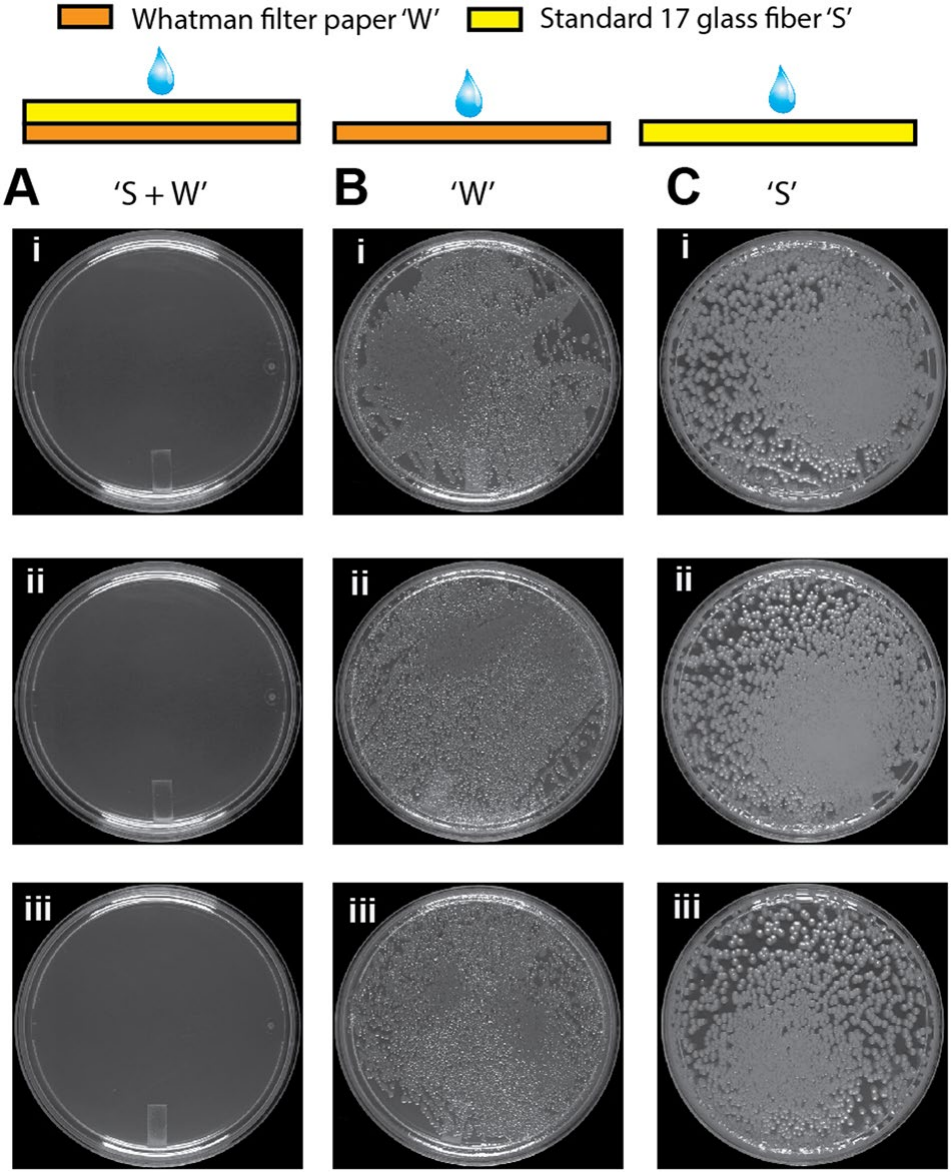
Dry storage of *E. coli* suspensions in paper stacks. (**A–C)** Culture results from *E*. *coli* suspensions dry stored in surface distributors: ‘S + W’ (**A**), single-layer Whatman filter paper, ‘W’ (**B**), and single-layer Standard 17 glass fibre, ‘S’ (**C**). A decontaminating reagent, NaOH, was dry stored in all devices. Surface distributors eliminated viable *E*. *coli* (**A**), while viable *E*. *coli* persisted in single-layer devices ‘W’ (**B**) and ‘S’ (**C**).

Sputum samples used for tuberculosis detection contain several non-*Mycobacterial* bacteria that must be killed before using the samples for *Mycobacterial* culture in a diagnostic lab^13^. Here, we demonstrate the ability of surface distributors to effectively decontaminate samples during dry storage in paper. *E*. *coli* was used as a model non-*Mycobacterial* bacterium. In the first experiment, a suspension of *E*. *coli* culture was introduced into three types of devices containing dried decontamination reagent, NaOH: (i) single layer Whatman filter paper only (‘W’), (ii) single layer Standard 17 only (‘S’), and (iii) a stack of Standard 17 placed on top of Whatman filter paper (‘S + W’). All paper layers were of size 8 cm × 2 cm and the fluidic capacities of ‘W’, ‘S’, and ‘S + W’ devices were 255, 640, and 895 µl, respectively. The optimal NaOH concentration for killing *E*. *coli*, leaving *Mycobacteria* viable, was determined to be 1% in separate experiments. For ‘W’ and ‘S’, 255 and 640 µl, respectively, of 1% NaOH was deposited into the devices and allowed to dry for 1 hour. For ‘S + W’, 255 µl of 3.5% NaOH was deposited and dried for 1 hour in the bottom filter paper layer before stacking the Standard 17 layer on top. This concentration was chosen such that when the ‘S + W’ device is rehydrated using an 895 µl solution, the effective concentration of NaOH in the rehydrated state would be 1%. After introduction of the bacterial suspension, the devices were dried and kept for 24 hours. After 24 hours, dried samples were rehydrated, centrifuged out of the devices, and used for culture. Because of effective mixing of dried NaOH with the bacterial suspension, there was no bacterial growth from samples stored in the surface distributors (Fig. 5A; N = 3). On the other hand, many colonies grew from samples dry stored in single layer Whatman filter paper (‘W’; Fig. 5B; N = 3) and single layer Standard 17 (‘S’; Fig. 5C; N = 3).

**Figure 6:**
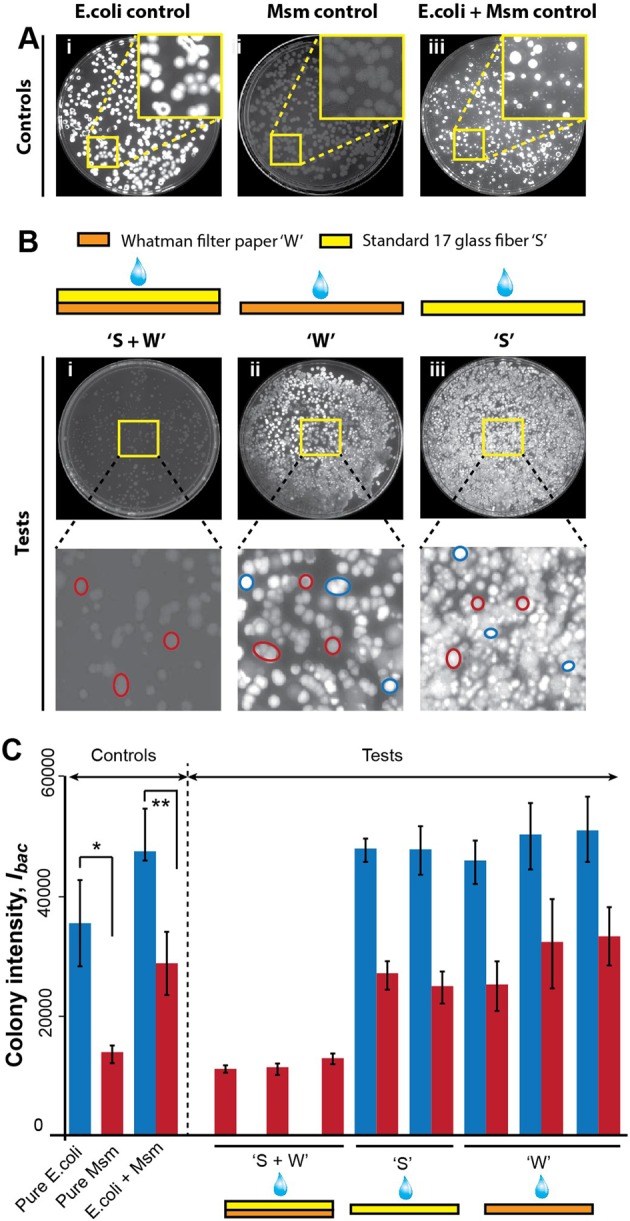
Decontamination of samples in paper stacks. (**A**) EtBr-stained control culture plates for pure *E*. *coli* (i), pure *Msm* (ii), and mixed *E*. *coli* + *Msm*. EtBr stains *E*. *coli* colonies brighter than *Msm*. (**B**) Culture results from *E*. *coli* + *Msm* suspensions dry stored in three types of test devices: surface distributors, ‘S + W’ (i), single-layer Whatman filter paper, ‘W’ (ii), and single-layer Standard 17 glass fiber, ‘S’ (iii). Representative low-intensity *Msm* colonies are highlighted in red and high-intensity *E*. *coli* colonies are highlighted in blue in the *insets*. (**C**) Quantitative fluorescence intensity analysis of EtBr-stained colonies. Control plates show higher mean intensities for pure *E*. *coli* than pure *Msm* cultures (*P < 10^−7^; N = 10), and the existence of colonies with both high and low intensities (**P < 10^−17^; N = 30) for mixed cultures. In test devices, only *Msm* were viable in ‘S + W’ devices (N = 3), indicating effective decontamination, while both *Msm* and *E*. *coli* were viable in ‘S’ (N = 2) and ‘W’ (N = 3).

The next experiment was identical to the previous experiment, except a suspension of *E*. *coli* and *Msm* was introduced into the three kind of devices. In order to distinguish colonies of *E*. *coli* from *Msm*, culture plates were fluorescently stained with EtBr; the LfrA efflux pump present in *Msm* results in reduced uptake of EtBr^14^. This was confirmed by control culture plates (Fig. 6A). When exposed to EtBr, *E*. *coli* colonies stained much brighter than *Msm* colonies (Fig. 6Ai,ii). When a mixed culture was stained, both brightly stained *E*. *coli* and less bright *Msm* colonies were observed (Fig. 6Aiii). When suspensions of *E*. *coli* and *Msm* were introduced into the three types of test devices, only the surface distributor devices effectively decontaminated the sample, killing all *E*. *coli* bacteria and leaving *Msm* viable (Fig. 6Bi), post drying and rehydration. For both single layer devices, ‘W’ and ‘S’, ineffective mixing between the decontaminating reagent and the bacterial suspension left viable *E*. *coli* colonies – mixed *E*. *coli* and *Msm* colonies were observed in both cases (Fig. 6bii,iii). Quantitative intensity analysis was further performed to confirm these findings. Control plates revealed that the mean fluorescence intensity of bacterial colonies, *I*_*bac*_, from pure *E*. *coli* cultures (~35,000 units) was significantly higher than that from pure *Msm* cultures (~14,000 units) (*P < 10^−7^; N = 10 colonies; Fig. 6C). For mixed cultures, *I*_*bac*_ of both types of colonies increased compared to pure cultures, but *I*_*bac*_ of *E*. *coli* (~47,000 units) continued to be significantly higher than that of *Msm* (~29,000 units) (**P < 10^−17^; N = 30 colonies; Fig. 6C). For surface distributors, *I*_*bac*_ of all detectable colonies was ~13,000 units, matching that of *Msm* pure culture controls (‘S + W’; Fig. 6C). For single layer devices, ‘S’, *I*_*bac*_ of the two colony populations were ~48,000 and 26,000 units (‘S’; Fig. 6C), and for single layer ‘W’ devices, they were ~49,000 and 30,000 units (‘W’; Fig. 6C). For both, the numbers matched the signatures of mixed *E*. *coli* + *Msm* controls.

### Enzymatic glucose detection assay

**Figure 7:**
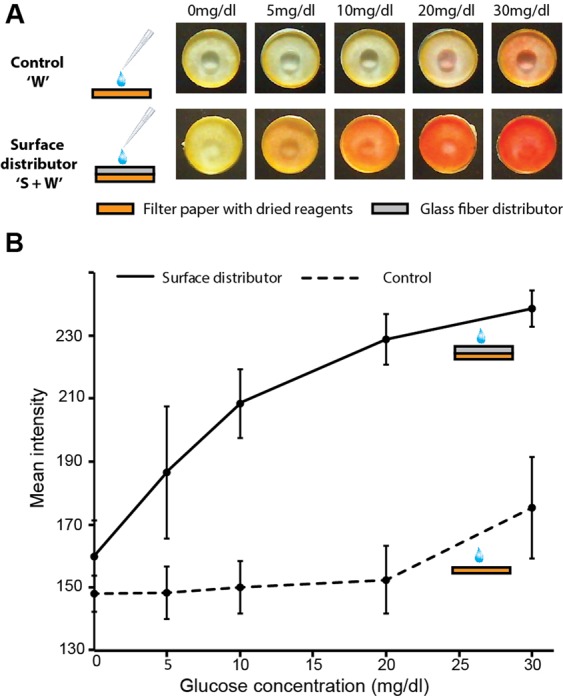
Colorimetric glucose detection in paper stacks. (**A**) Snapshots of two kinds of glucose detection devices: single layer filter paper (control) and a stack of a glass fiber distributor placed on top the filter paper (surface distributor), at *t* = 4 min after sample addition, for various glucose concentrations. (**B**) Plot of mean signal intensities at *t* = 4 min after sample addition for controls (dashed line) and surface distributors (solid line) for various glucose concentrations. Surface distributors enabled more sensitive glucose detection compared to single-layer controls.

Uniform rehydration and effective mixing enabled by surface distributors can also improve the performance of colorimetric paper-based analytical devices, as demonstrated here in the context of an enzymatic glucose detection assay. Reagents required for glucose detection were dry stored in a disc of Whatman filter paper and rehydrated with a glucose solution, either directly (‘W’; Fig. 7A), or through a Standard 17 surface distributor (‘S + W’; Fig. 7A). In the presence of glucose, the enzymatic reaction yields pink-colored products. For direct rehydration in a single layer device, pink color was not observed for 0, 5, and 10 mg/dl, but a slight pink color was observed for 20 and 30 mg/dl (‘W’; Fig. 7A). Further, the difference in intensities between absence (0 mg/dl) and presence of glucose was only statistically significant for 30 mg/dl (P = 0.05; N = 3). Whereas when rehydrated through a surface distributor, pink color was observed for 5 mg/dl and at all higher concentrations (‘S + W’; Fig. 7A). The difference in intensities between absence and presence of glucose was statistically significant for 10 mg/dl (P < 0.01; N = 3) and higher concentrations. Mean pink color intensities as a function of glucose concentration for the two types of devices are plotted in Fig. 7B. Surface distributors, thus, significantly enhanced the sensitivity compared to single layer paper devices, as are traditionally used for diagnostics. A significant factor in improving the sensitivity may be the additional fluid volume that was accommodated by the surface distributor (57 µl) compared to a single filter paper layer (12 µl).

Of further note is the uniformity of pink color generated over the 1 cm^2^ area of the paper sensor when used with a surface distributor. Colorimetric signals generated in traditional µPADs are associated with steep gradients with standard deviations in pixel intensities ranging from 34 to 40%^15^, which makes it difficult to interpret the signal^7^. Recently, it was shown that these gradients arise as a result of wicking fluid pushing small colorimetric molecules to the edge of the detection zones^15^. No obvious steep chemical gradients were observed in our glucose sensors utilizing surface distributors, despite using relatively large fluid volumes (57 µl) and sensor surface areas (1 cm^2^).

## Discussion

The paper microfluidic device designs presented here address the largely unsolved problem of non-uniform rehydration of dried reagents in paper analytical and storage devices. Uniformity of rehydration and the ensuing enhancement in interaction between the rehydrating fluid and dried reagents has been enabled by novel paper architectures that have not been presented before. Parallel assemblies of paper materials that differ in flow properties like permeability and capillary pressure can result in interesting and non-obvious flow phenomena, which have been exploited in this work. Parallel paper assemblies have previously been used as tunable delay shunts^16^. More recently, an assembly of a duct in parallel with a paper channel has been shown to enhance the rate of wicking^17,18^. However, this is the first report of the use of parallel paper assemblies for uniform rehydration.

The new design concepts described here present new opportunities for the development of sample collection and storage devices. While the use of dried blood spot (DBS) cards for collection and transport of blood was first described in the 1860s^19^, there has been surprisingly little technological development in this field since. The DBS card technology is known to have certain inherent limitations, e.g. formation of non-uniform spots and spatial heterogeneity in concentration of analytes over the spot^19^. In addition, a major limitation of DBS cards is their limited fluidic capacity (typically ~100 µl). This prevents their use in collection of other body samples like sputum, saliva, urine etc., in which concentrations of analytes may be low enough to warrant collection of larger specimen volumes. For example, the detection of tuberculosis from sputum requires a limit of detection of ~100 bacteria/ml of fluid^20^; collection of a few drops of specimen is not sufficient. If it were possible to dry store large volumes of sputum samples (>1 ml) collected at remote locations so that the samples remained stable over several weeks, it would tremendously aid in increasing the penetration of tuberculosis (TB) diagnosis into remote locations. For this, devices that can accommodate larger fluid volumes and uniformly mix them with sample stabilization agents are needed. The technology presented here fills this gap. The challenge of having to dry a potentially infected specimen in open air remains. To address this, our future work includes designing enclosures containing desiccants, so paper stacks containing specimens may be dried in sealed containers.

## Methods

### Chemicals

#### For drying on paper

Orange-red food dye was obtained from Ajanta Industries (Gurugram, India); papers were dipped in a solution of 2 g/L and dried in an incubator at 37 °C; *for preparation of mock sputum*: methylcellulose (M-0262) and egg yolk emulsion (17148) were obtained from Sigma-Aldrich; mock sputum was prepared as a solution of 1.8% w/v methylcellulose and 10% egg yolk emulsion in DI water^21^; *for Mycobacterial culture*: Tryptone soya broth 24392 (TM 018) was obtained from SRL and Middlebrook 7H9 Broth Base (M198-500G) was obtained from HiMedia. Tween-80 28940 (1628157) and glucose 51758 (0449130), obtained from SRL, were used as supplements; *for E*.*coli culture*: Luria Bertani broth, Lennox 14593 (LM 021) and agar powder 85473 (014042) were obtained from SRL; *for staining bacterial colonies*: ethidium bromide (EtBr) was obtained from SRL; *for glucose detection assay*: dextrose (AR 51758) was obtained from SRL and the following reagents were obtained from Sigma: glucose oxidase (G2133), horseradish peroxidase (P8375), 4-amino antipyrine (A4382) and 2,4,6-tribromo-3-hydroxybenzoic acid (439533).

### Bacterial cultures

*Mycobacterium smegmatis mc*^2^ 155 (*Msm*) and *E*. *coli* K-12 (MG1655) were obtained as kind gifts from Prof. Deepak Saini and Prof. Jayant Modak, respectively (Indian Institute of Science, Bangalore). The *Msm mc*^2^ 155 was routinely grown in Tryptone soya broth with 0.05% Tween 80 for 24 hours at 37 °C, 180 RPM (primary culture) followed by growth in Middlebrook 7H9 broth with 2% glucose for 24 hours at 37 °C, 180 RPM (secondary culture). *E*. *coli* K-12 was cultured overnight in Luria Bertani broth at 37 °C at 90 RPM.

### Materials for fabrication

Standard 17 glass fiber, Whatman filter paper Grade 1, and nitrocellulose (FF120HP) membranes were acquired from GE Healthcare Life Sciences (Bangalore, India). Supporting plastic materials: 0.18 mm-thick transparency sheets and 2.15 mm-thick transparent acrylic sheets were acquired locally. A double-coated pressure-sensitive adhesive film (PSA; 3M^TM^ 9731) was used to secure stacked paper and plastic layers. All materials were cut using a 50 W CO_2_ laser in a VLS 3.60 laser engraver (Universal Laser Systems, Scottsdale, AZ). All designs were created in AutoCAD (Autodesk, San Rafael, CA).

### Imaging setup and image analysis

All flow and imaging experiments were carried out in a custom-made dark box equipped with white LED lights. To enable imaging from underneath, devices were placed on an elevated acrylic stage at an elevation of 15 cm from the base. Time-lapse images of flow were acquired using a webcam (C525; Logitech, Newark, CA) secured on the base, operated using HandyAvi (AZcendant, Tempe, AZ) software. Webcam capture settings were maintained constant for all experiments. All experiments were conducted by keeping a control device (single-layer) next to a test device (stacked layers) and imaging both devices in the same frame to minimize run-to-run variation in lighting conditions, if any. Images were analyzed using ImageJ; after splitting color channels, the red channel was used for analyzing images of devices containing orange dye and blue channel for images of devices containing glucose-detection reagents. For rectangular paper membranes, linear intensity profiles, *I*(*x*) (0 < *x < *8 cm) were obtained by averaging intensities along the width at successive lengths. To account for non-uniform lighting over the area of the device, the profiles were first corrected as follows. Assuming that the dye was uniformly dried over the surface before rehydration, the ideal intensity profile at *t* = 0 s (before fluid introduction) would be a straight horizontal line. It was assumed that the magnitude of this horizontal line would equal *I*_*max*_, the maximum intensity value in *I*(*x*) at *t* = 0 s. Deviations from this horizontal line caused by non-uniform lighting were quantified by fitting a third order polynomial, *F*(*x*) (0 < *x* < 8 cm), to the profile obtained at *t* = 0 s for each device. The intensity correction function for each device was then calculated as *F*_*correction*_(*x*) = *I*_*max*_ /*F*(*x*). The final intensity profile *I*(*x*) after complete rehydration was then multiplied by *F*_*correction*_(*x*) to eliminate the effect of improper lighting. Area under the curve of corrected linear intensity profiles was calculated using Simpson’s one-third rule. All statistical comparisons were made using two-tailed Student’s t-tests conducted in Excel (Microsoft, Redmond, WA).

Bright field and fluorescent images of EtBr-stained bacterial culture plates were obtained using a gel documentation system (UVITEC Essential V6). The average fluorescence intensity of bacterial colonies, *I*_*bac*_, was obtained using ImageJ. Ten colonies were chosen randomly. An oval region of interest was created around each colony and its average intensity was measured. The average intensities of 10 such colonies were further averaged to evaluate *I*_*bac*_.

### Fluid and membrane characterization

Viscosity of mock sputum was measured using a cone and plate rheometer MCR 301 (Anton Paar, Graz, Austria). Scanning electron microscope (SEM) images of gold-sputtered membranes were acquired using a Zeiss Ultra 55 field emission SEM. The 4-cm wicking time, *T*_*4-cm*_, for membranes was obtained using a custom-made humidity chamber in which 1 cm-wide (FF120HP and Whatman filter paper) or 0.5 cm-wide (Standard 17) membranes were laid horizontally attached to a fluid reservoir. Water was introduced in the reservoir at *t* = 0 and time-lapse images were acquired using a webcam. *T*_*4-cm*_ is reported as mean ± standard deviation. The Washburn coefficient, *K*, was calculated assuming that the fluid fronts follow the Washburn relationship, $L=K\sqrt{t}$, where $L$ is distance of the fluid front from the source and $t$ is time^16^. Substituting *L* = 4 cm and *t* = *T*_*4-cm*_ in this equation enabled evaluation of *K*. The capillary pressure of Standard 17 and Whatman filter paper was determined as a function of saturation (fraction of fluid-filled pores) using a method described by Rath *et al*.^22^ that involved centrifugation of a 1 cm × 1 cm membrane at successively higher speeds and measuring the weight of fluid retained in the membrane.

### Bacterial decontamination in paper stacks

Two sets of experiments were conducted – the first involving a suspension of *E*. *coli* only (10^4^ CFU/ml in Milli-Q water), and the second containing a suspension of *E*. *coli* and *Msm* (10^2^ CFU/ml *E*. *coli* and 10^2^ CFU/ml *Msm* in Milli-Q water). Bacterial suspensions were introduced into devices from the top and center. The volumes were chosen to match the fluidic capacities of each device. Following introduction of bacterial suspensions, devices were dried and stored for 24 hours at room temperature, after which, they were rehydrated with equal volumes of water and put into 50-ml Corning tubes for removal of the bacterial suspension by centrifugation at 9000 rpm (3170 rcf) for 5 minutes. The obtained suspensions were plated (100 µl/plate) and incubated at 37 °C for 48 hours before colony counting. For experiments involving a mixture of *E*. *coli* and *Msm*, 48-hour culture plates were stained by introducing a 500 µl solution of 0.1 mg/ml EtBr in each plate, incubated at room temperature for 5 min, followed by fluorescence imaging.

### Enzymatic glucose detection assay

The bienzymatic assay for glucose testing, adapted from assays described by Santana-Jimenez *et al*.^23^ and Zhu *et al*.^24^, involved glucose oxidase (20 mg/ml), horseradish peroxidase (1 mg/ml), 4-amino antipyrine (20 mg/ml) and 2,4,6-tribromo-3-hydroxybenzoic acid (5 mg/ml). Equal volume of each reagent was mixed to make a master mix. Two types of devices were tested: (i) single-layer Whatman filter paper only (‘W’) and (ii) a stack of Standard 17 placed on top of Whatman filter paper (‘S + W’). All paper layers were circles of 1 cm^2^ area (~1.13 cm dia). Fluidic capacities of ‘W’ and ‘S + W’ were 12 µl and 57 µl, respectively. Master mix (12 µl) was added to filter paper discs and the reagent-containing discs were dried in an incubator at room temperature (25 °C) for 2 hours before device assembly. Different concentrations of glucose solutions (0, 5, 10, 20, and 30 mg/dl) were prepared by dissolving dextrose in water. ‘S + W’ devices were rehydrated with 57 µl and ‘W’ devices with 12 µl of the respective glucose solutions and colorimetric results were recorded at 240 s from glucose addition.

## Supplementary information

Compiled Supplementary Info

## Data Availability

All data generated or analyzed during this study are included in this published article (and its Supplementary Information files).
