# Supplementary material for: Paper Stacks for Uniform Rehydration of Dried Reagents in Paper Microfluidic Devices
Source: Sci Rep. 2019 Oct 31;9:15755. doi: 10.1038/s41598-019-52202-9 (PMC6823543; doi:10.1038/s41598-019-52202-9)
Supplement: Supplementary file 1 — Compiled Supplementary Info [file 41598_2019_52202_MOESM1_ESM.pdf]

Paper Stacks for Uniform Rehydration of Dried Reagents in Paper Microfluidic Devices

Debayan Das[1], Andrea Dsouza[1], Navjot Kaur[1], Shruti Soni[1], Bhushan J. Toley[1*]

[1] Department of Chemical Engineering
Indian Institute of Science
Bangalore, India
560012

Keywords: MicroPADs, dry blood spot cards, global health, paper-based microfluidics, wicking flow, tuberculosis diagnostics, sputum storage

*Correspondence to:
Bhushan J. Toley
Department of Chemical Engineering
Indian Institute of Science
Bangalore, India, 560012
Phone: +91-80-22933114
Email: bhushan@iisc.ac.in

Supplementary Information

Supplementary Figure S1. Schematics of various device designs

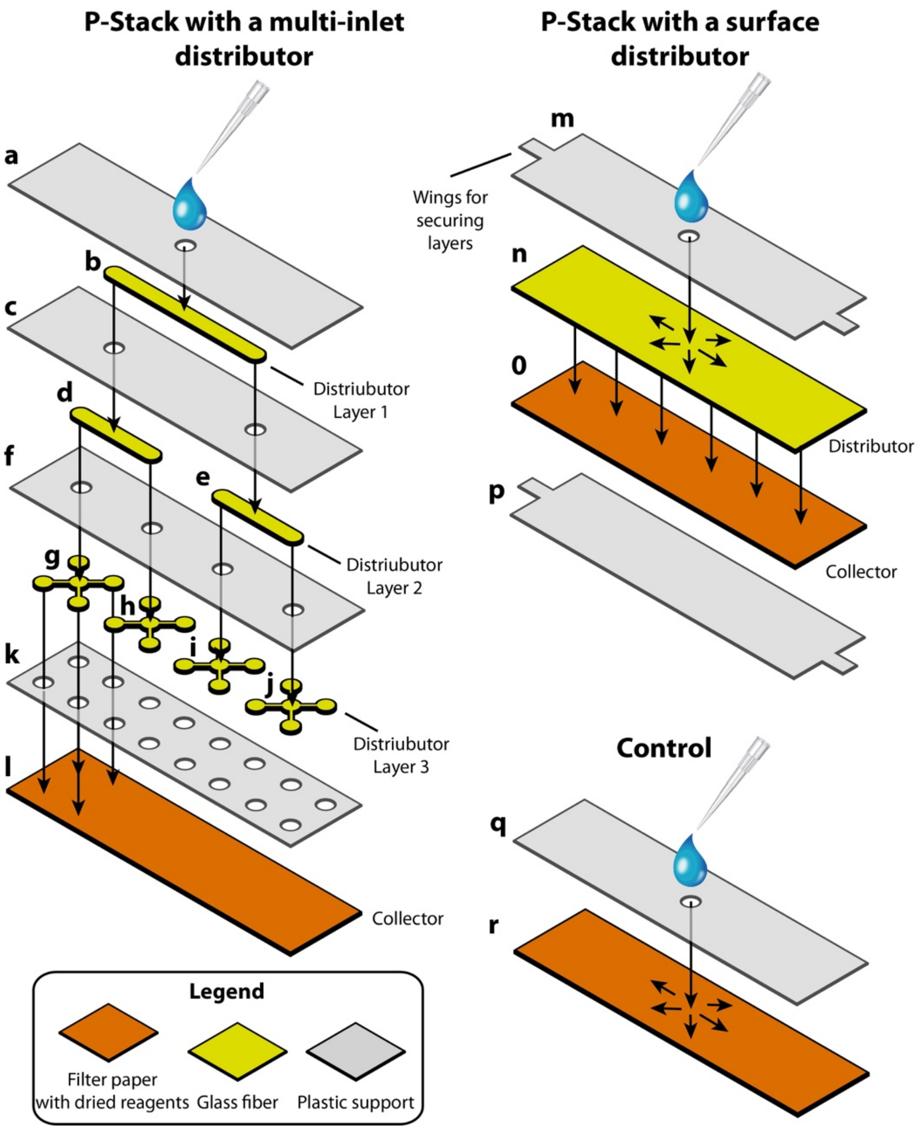

Supplementary Figure S1. Schematics of the various designs showing individual parts.

Detailed part drawings of some key components are provided. In Figure S3 individual parts are labeled using alphabets **a** through **r**. Figure S4 contains the corresponding part drawings. The dimensions of some parts are obvious and those parts are not included in Figure S4.

Supplementary Figure S2. Detailed part drawings

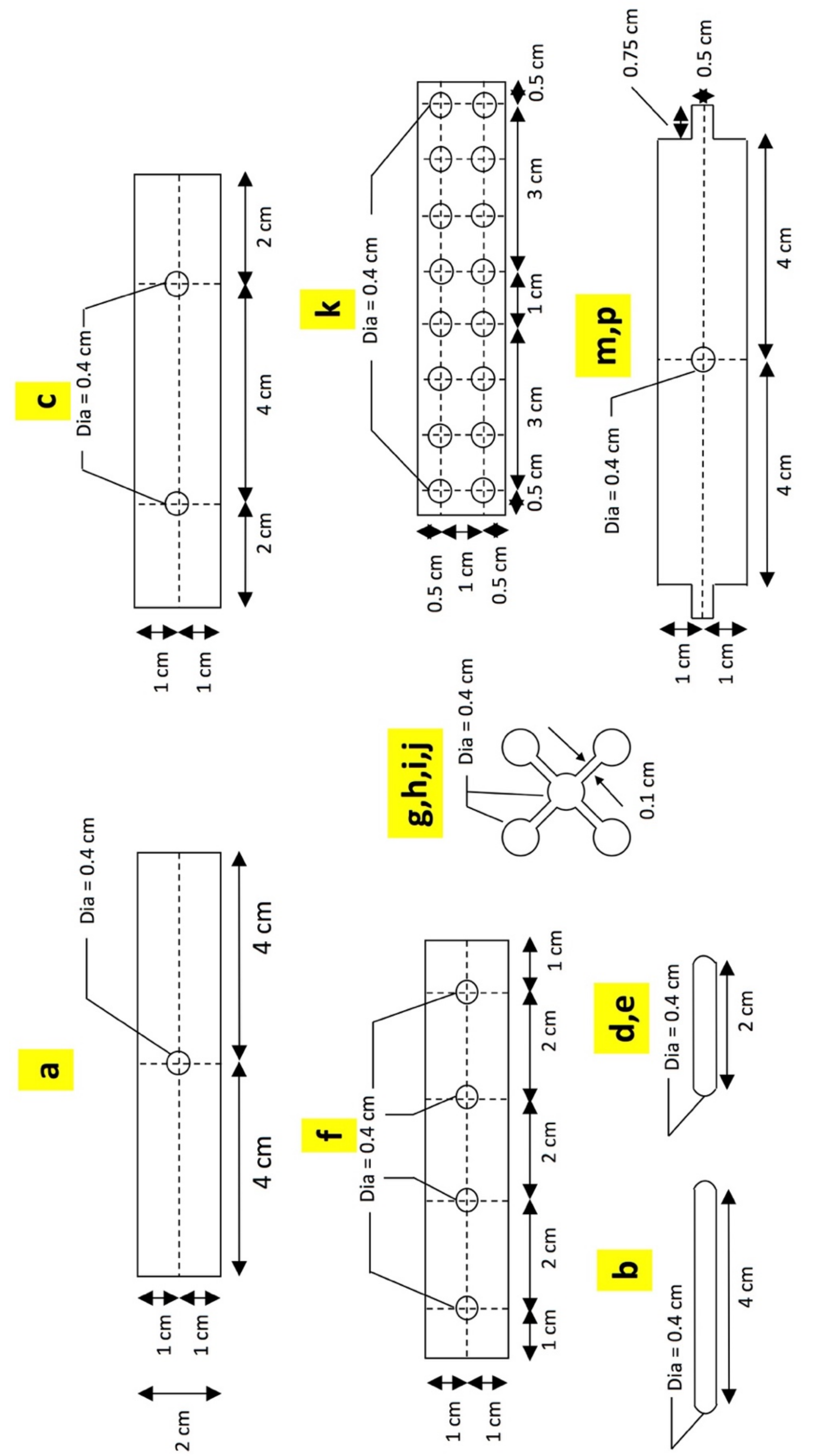

Supplementary Figure S2. Drawings and dimensions of important parts. Alphabet labels correspond to the labels in Supplementary Figure S3.

Supplementary Figure S3: Multi-inlet distributors

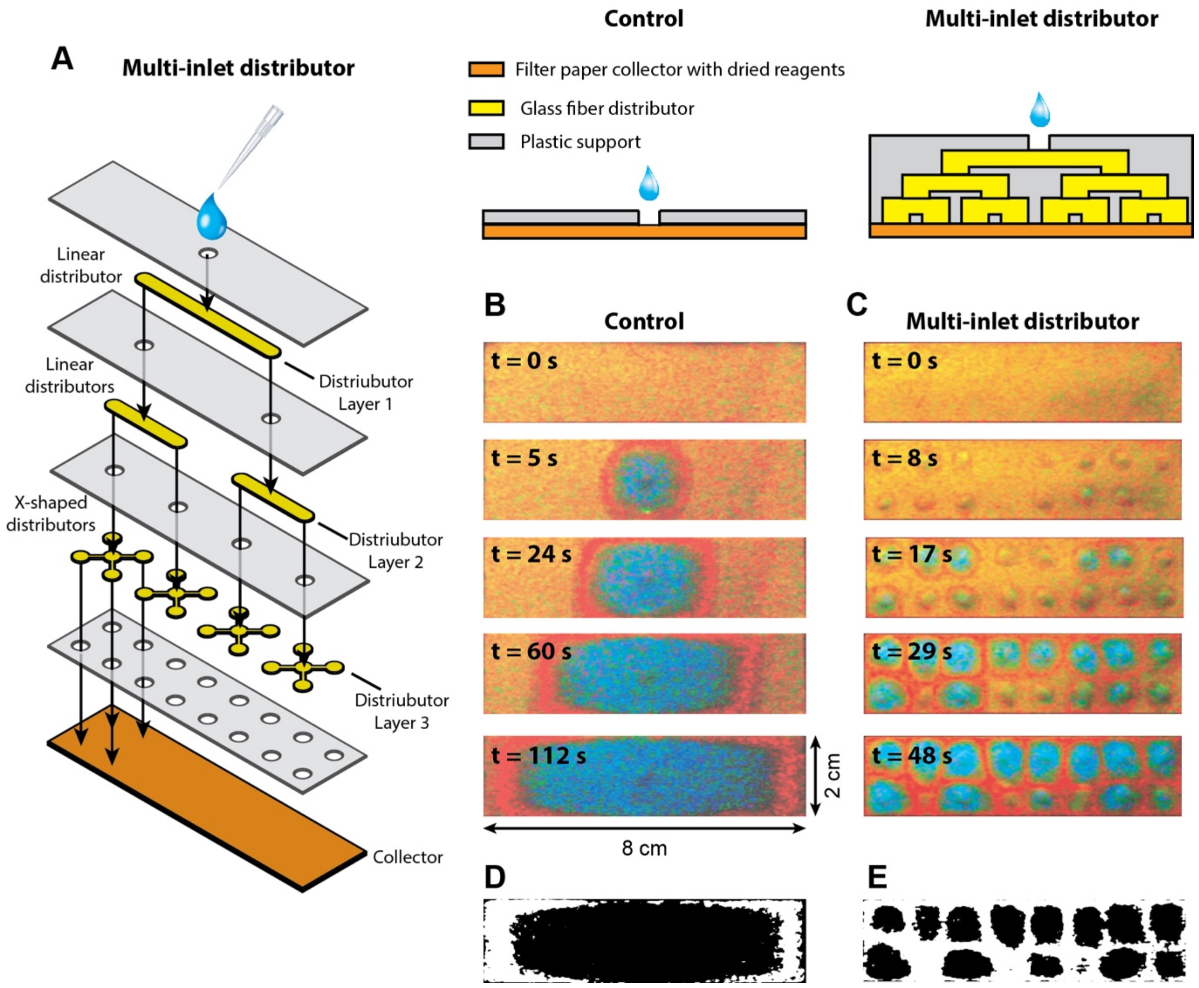

Supplementary Figure S3. Multi-inlet distributors **A**. The multi-inlet distributor distributes fluid into many exit points from which it enters the collector. **B**. Fluid entering a single layer filter paper membrane (control) rehydrates the dried orange dye and pushes it to the edges. **C**. The multi-inlet distributor splits incoming fluid into 16 closely spaced exit points and reduces migration of the rehydrated orange dye to the edges. **D-E**. Thresholded images showing areas containing orange dye as white and areas lacking orange dye (non-reactive areas) as black for control (**D**) and the multi-inlet distributor (**E**). The multi-inlet distributor significantly reduced the non-reactive area ($P<0.005$; $N=3$).

In conventional paper microfluidic devices, fluids wick into regions containing dried reagents from a single inlet point. We hypothesized that increasing the number of inlet points using appropriate distributors would reduce the distance over which rehydrated reagents could be displaced and enhance interaction between the dried reagents and rehydrating fluid. To test this hypothesis, we took motivation from multi-layer three-dimensional paper analytical devices (3D µPADs) that are designed to distribute a given fluid volume into multiple analytical zones[1].

Multiple layers of paper and plastic were stacked such that each distributor layer increased the number of fluidic exit points by a factor of 2 (linear distributor) or 4 (X-shaped distributor; Fig. S3A). The specific implementation shown in Fig. S3A consisted of three distributor layers 1, 2, and 3 (yellow; Fig. S3A) that increased the number of exit points by 2x, 2x, and 4x, respectively, thus splitting fluid entering from one inlet into 2 x 2 x 4 = 16 outlets, uniformly spread over a filter paper membrane containing dried reagents (orange; Fig. S3A). Each plastic supporting layer (grey; Fig. S3A) consisted of a transparency sheet backed with double-sided PSA from both sides. In this implementation, all distributor layers were fabricated with Standard 17 glass fiber and the bottom 'collector' layer containing dried reagents was fabricated with Whatman filter paper. These membranes were chosen because Standard 17 has a lower capillary pressure compared to filter paper (see Figure 2 in main manuscript) and therefore readily releases fluid into the filter paper collector.

Rehydration of a dried colored dye using multi-inlet distributors was compared to single-layer paper membranes (controls). The devices consisted of large (8 cm x 2 cm) filter paper membranes impregnated with dried food coloring dye. Detailed dimensions of the device are provided in Fig. S1 and Fig. S2. The control, which contained only a single filter paper membrane, was rehydrated with 255 µl DI water corresponding to the fluidic capacity of the membrane. Fluid added at the center of the membrane wicked into the membrane in all directions away from the source. As the fluid front advanced, the dye dissolved in the water and moved along with the front (Fig. S3B). The membrane was fully wet at 112s, at which point, a significant portion of the dye was pushed to the edges producing a large island devoid of the dye (Fig. S3B). In comparison, the multi-inlet distributor distributed fluid into 16 outlets of diameter 4 mm each (Fig. S3C). A total of 550 µl DI water was used corresponding to the fluidic capacity of the multiple layers. The filter paper layer was fully wet in 48s (Fig. S3C).

The reduction in rehydration time was a result of reduced wicking distances in the collector because of the multiple entry points and minimal additional resistance to flow provided by the glass fiber distributor composed of large pores. At 48s, rehydrated dye was present throughout the 16 cm^2 area, although large islands of dye-free regions were observed (Fig. S3C). Regions devoid of dye correspond to regions where the rehydrating fluid cannot interact with the dried reagents (dye) and constitute a 'non-reactive' area. Therefore, a better rehydrating strategy would be the one that minimizes the non-reactive area or enhances the 'reactive area'. The percentage non-reactive area, estimated by thresholding the end-point images (Fig. S3D,E), reduced from a mean value of 70.4% for controls to 46.7% for multi-inlet distributors ($P<0.005$; $N=3$). The uniformity of the rehydrated dye was far from ideal, apparent from the inconsistent size and shape of the 16 dye-free regions formed at the inlets (Fig. S3C,E). Despite the lack of uniformity, a statistically significant reduction in the non-reactive area suggested that increasing the number of inlet points aids in enhancing the interaction between the dried reagents and the rehydrating fluid. Note that for 3D μPADs, techniques have been developed specifically to improve uniformity of distribution[2] but that was not the objective of this work. The objective of this work was to test the hypothesis that increasing the number of fluid inlet points into the collector containing dried reagents reduces the non-reactive area, which was tested and found to be true.

Supplementary Figure S4. Effect of the type of collector membrane on surface distributors

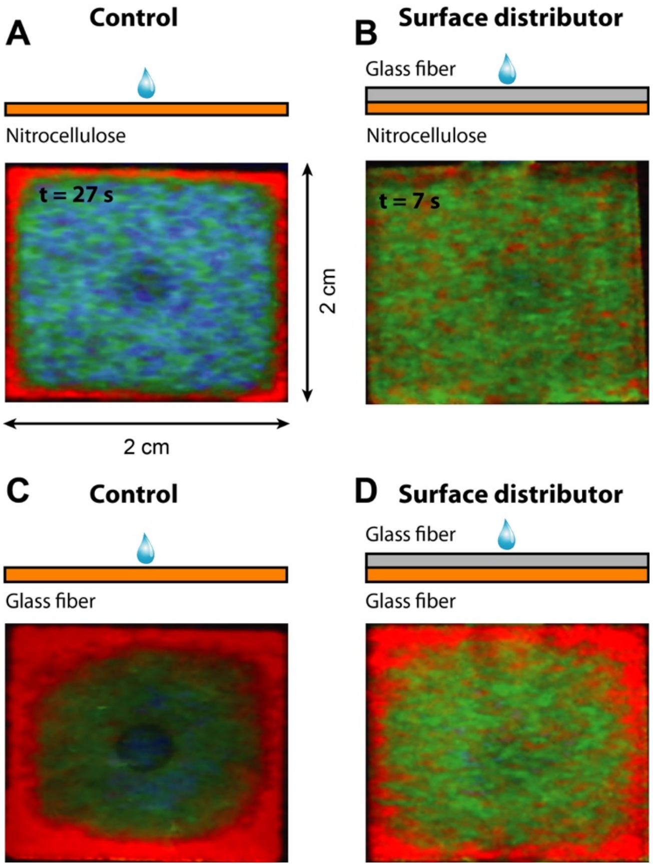

Supplementary Figure S4. Effect of the collector material membrane on surface distributors. Collectors were constructed from nitrocellulose (**A-B**) and Standard 17 glass fiber (**C-D**). Standard 17 was used as the distributor in all cases. For nitrocellulose, the surface distributor significantly improved the uniformity of rehydration (**A-B**). For Standard 17, there was no significant improvement (**C-D**).

The possibility of using materials other than filter paper as the collector was briefly explored using two alternatives – nitrocellulose (NC) FF120 and Standard 17 glass fiber. In both cases, the distributor was made of Standard 17 glass fiber and controls without distributors were used for direct comparison. Collector and distributor membranes were constructed as 2 cm x 2 cm squares (Fig. S4). When the NC control was rehydrated from the center, the rehydrated reagents were efficiently pushed to the edges (Fig. S4A), whereas this compression effect was completely eliminated using the surface distributor (Fig. S4B). Compared to the control, the rehydration time was reduced from 27s to 7s (Fig. S4A-B). On the other hand, when Standard 17 was used as the collector, there was not much difference in the rehydration pattern between

the control (Fig. S4C) and the surface distributor (Fig. S4D) and non-reactive zones were formed in both cases. Rehydration times were ~10s for both the control and the distributor. As stated in the main article, surface distributors work effectively only when there is a significant difference in the wicking rates of the distributor and collector membranes. Measured values of the parameter $T_{4\text{-}cm}$ for Standard 17 and NC FF120 were 11.3s ± 1.15s and 148s ± 10s, respectively. This large difference ensures effective functioning of the surface distributor with a NC collector and Standard 17 distributor. Whereas when Standard 17 is used both as the collector and distributor, there is no difference in wicking rates, so the stack acts as one single thick membrane and reagents continue to get pushed to the edges (Fig. S4D).

References

1. Martinez, A. W., Phillips, S. T. & Whitesides, G. M. Three-dimensional microfluidic devices fabricated in layered paper and tape. *Proc. Natl. Acad. Sci. U. S. A.* **105**, 19606–19611 (2008).
2. Morbioli, G. G., Mazzu-nascimento, T., Milan, L. A., Stockton, A. M. & Carrilho, E. Improving Sample Distribution Homogeneity in Three-Dimensional Micro fl uidic Paper-Based Analytical Devices by Rational Device Design. 6–12 (2017). doi:10.1021/acs.analchem.6b04953
3. Rath, D., Sathishkumar, N. & Toley, B. J. Experimental Measurement of Parameters Governing Flow Rates and Partial Saturation in Paper-Based Microfluidic Devices. *Langmuir* **34**, 8758–8766 (2018).
